# Anatomical entity mention recognition at literature scale

**DOI:** 10.1093/bioinformatics/btt580

**Published:** 2013-10-25

**Authors:** Sampo Pyysalo, Sophia Ananiadou

**Affiliations:** National Centre for Text Mining and University of Manchester, Manchester, UK

## Abstract

**Motivation:** Anatomical entities ranging from subcellular structures to organ systems are central to biomedical science, and mentions of these entities are essential to understanding the scientific literature. Despite extensive efforts to automatically analyze various aspects of biomedical text, there have been only few studies focusing on anatomical entities, and no dedicated methods for learning to automatically recognize anatomical entity mentions in free-form text have been introduced.

**Results:** We present AnatomyTagger, a machine learning-based system for anatomical entity mention recognition. The system incorporates a broad array of approaches proposed to benefit tagging, including the use of Unified Medical Language System (UMLS)- and Open Biomedical Ontologies (OBO)-based lexical resources, word representations induced from unlabeled text, statistical truecasing and non-local features. We train and evaluate the system on a newly introduced corpus that substantially extends on previously available resources, and apply the resulting tagger to automatically annotate the entire open access scientific domain literature. The resulting analyses have been applied to extend services provided by the Europe PubMed Central literature database.

**Availability and implementation:** All tools and resources introduced in this work are available from http://nactem.ac.uk/anatomytagger.

**Contact:** sophia.ananiadou@manchester.ac.uk

**Supplementary Information:** Supplementary data are available at *Bioinformatics* online.

## 1 INTRODUCTION

During the past two decades, there has been increasing research interest in the development of resources and methods for automatically processing the biological and medical scientific literature to address the multiple challenges created by its size and increasingly rapid growth. Annotated resources and tools targeting various aspects of scientific texts have been introduced, including, for example, mentions of gene names (Smith *et al.*, 2008), chemicals (Kolluru *et al.*, 2011) and organisms (Gerner *et al.*, 2010), relations such as protein–protein interactions (Krallinger *et al.*, 2007), drug–drug interactions (Segura-Bedmar *et al.*, 2011) and molecular events and biological processes such as gene expression, protein phosphorylation, viral infection and angiogenesis (Kim *et al.*, 2011; Pyysalo *et al.*, 2012a, b).

**Fig. 1.**
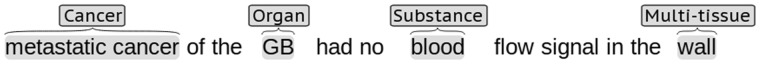
Annotation example showing tagged anatomical entity mentions

Much of recent work in biomedical natural language processing and text mining has focused on molecular level entities and processes, addressing e.g. the detection of gene name mentions and protein–protein interaction statements. However, a comprehensive analysis of biomedical text requires automatic systems to address entities also at other levels of biological organization. In this study, we focus on the development and large-scale application of a machine learning-based system on for the recognition of mentions of *anatomical entities*, organism parts at levels of organization between the molecular and the whole organism (Fig. 1). Building on a state-of-the-art entity mention tagger based on conditional random fields, we implement and evaluate a variety of approaches for improving mention recognition performance. For training and evaluation, we extend existing corpora through manual annotation to create a large, open-domain, cross-species resource covering both publication abstracts and full-text documents.

The developed integrated system, AnatomyTagger, can be applied either as a standalone tagger, a component in pipelines based on the Unstructured Information Management Architecture (UIMA) (Ferrucci and Lally, 2004; Kontonatsios *et al.*, 2013) or as a web service, and complements existing tools for the recognition of molecular entities and whole organism mentions to facilitate comprehensive analysis of entity references in biological and medical text. To further advance such analysis and to demonstrate the feasibility of large-scale tagging, we apply the system to all available open access domain publications, recognizing 48 million anatomical entity mentions. The contribution of this study is thus 4-fold: the introduction of a new integrated corpus, the detailed evaluation of multiple methods and resources for anatomical entity tagging, the implementation of a new system incorporating the best-performing approach to tagging and the application of this tagger to the entire open-access literature. All contributed tools and resources are made available under open licenses.

## 2 APPROACH

We next present the ontological basis, task setting and basic system architecture applied in our work.

### 2.1 Ontological basis and entity types

We define the target of our analysis, anatomical entity mentions, primarily with reference to the Common Anatomy Reference Ontology (CARO) (Haendel *et al.*, 2008) and the Foundational Model of Anatomy (FMA) (Rosse and Mejino, 2003). CARO is a small species-independent upper-level ontology proposed to unite species-specific ontologies such as the extensive human-specific FMA that it is based on. In defining the annotation scope and types, we follow our previous work (Ohta *et al.*, 2012), in particular in annotating also pathological parts of organism anatomy—important in discussions of real-world, as opposed to idealized canonical anatomy—and excluding from scope two classes of entities, whole organisms and biological macromolecules, whose recognition in text has been extensively studied in previous work (e.g. Smith *et al.*, 2008; Gerner *et al.*, 2010). We additionally differentiate cancers as a specific subtype of pathological formations, giving the types presented with examples in Table 1.

**Table 1.** Entity types and implicit ontological structure

Type	Examples
Anatomical entity*^a^*	cell, heart, blood
Anatomical structure*^a^*	cell, heart, head
Organism*^b^*	human, drosophila
Organism subdivision	head, limb, hand
Anatomical system	vascular system
Organ	liver, heart, lung
Multi-tissue structure	artery, cornea
Tissue	epithelium, bone
Cell	epithelial cell
Developing anatomical structure	embryo, fetus
Cellular component	nucleus, plasmid
Biological macromolecule*^b^*	cyclin, insulin
Organism substance	blood, serum, urine
Immaterial anatomical entity	lumen, bone cavity
Pathological formation	wound, ulcer, edema
Cancer	tumor, carcinoma

*Note*: Indentation corresponds to *is-a* structure. *^a^*Not annotated: implicit structure only. *^b^*Not annotated: out of scope.

### 2.2 Task setting

We formalize the basic task as identifying all contiguous non-overlapping sequences of characters that refer to anatomical entities in unstructured text and assigning each such mention exactly one type from a given set of disjoint upper-level ontological categories that jointly cover all targeted entities (Table 1). As mentions are contiguous and non-overlapping and the assigned types disjoint, mention detection and classification can be cast as a standard sequential labeling task, where each token is identified e.g. as (B)eginning, (I)nside or (O)utside an entity mention, with the former two tag categories additionally identifying the entity type (e.g. B-Cell). The basic task is addressed as supervised sequential labeling using this representation.

### 2.3 Architecture

**Fig. 2.**
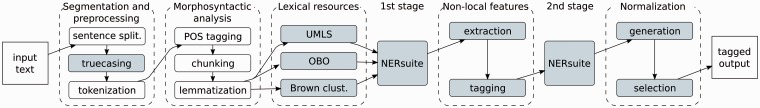
AnatomyTagger architecture. Parallel arcs indicate mutually independent stages of processing. Processing stages drawn shaded involve models or tools newly introduced in this study

The developed system has the pipeline architecture illustrated in Figure 2. Broadly, input text is first segmented into sentences and tokens, lexical processing and shallow syntactic analysis is then performed to assign features such as part-of-speech (POS) tags and additional features are then generated through lookup against various lexical resources. Entity mention recognition and classification is then performed in two stages, with the second stage incorporating non-local features derived from the first-stage analysis. The following section details the methods and resources applied to implement, train and evaluate the system.

## 3 METHODS

We base the primary machine learning components of our method on NERsuite (http://nersuite.nlplab.org/), a retrainable named entity recognition (NER) toolkit building on the CRFsuite (Okazaki, 2007) implementation of Conditional Random Fields (CRFs) (Lafferty *et al.*, 2001). CRFs are frequently applied to sequential labeling tasks such as entity mention recognition and are at the core of numerous state-of-the-art taggers for various tasks. The following sections detail the processing applied for feature generation, the application of the machine learning method and other stages of the AnatomyTagger pipeline.

### 3.1 Text segmentation and preprocessing

The applied machine learning method operates on a token basis and makes use of information regarding the relative position of tokens within sentences. We thus initially segment input text into sentences and those further into tokens. For sentence segmentation, we apply the GENIA sentence splitter trained on the GENIA treebank (Tateisi and Tsujii, 2006) with a heuristic post-processor to correct some common errors. To allow the tagger to assign mention boundaries at a fine granularity (e.g. in words such as ‘platelet-derived’), we apply an aggressive tokenization strategy that preserves contiguous sequences of alphanumeric characters as tokens and assigns all other single characters into separate tokens.

We additionally consider the effect of *truecasing*, generally defined as the process of restoring the correct case to text with missing (or incorrect) case information (Lita *et al.*, 2003). In the context of processing text with (largely) correct case, such as scientific publications, we understand truecasing to involve restoring ‘neutral’ case to (i) all sentence-initial (capitalized) words, (ii) words in Title Case in the titles of articles, sections, tables and so forth and (iii) words in ALL-UPPER case contexts such as some publication titles. Given the limited scope of this task compared with all-words truecasing and the comparative ease of the task, we opted here to implement basic unigram-based truecasing, using a model derived from a random sample of the 2012 PubMed® baseline distribution. Truecasing is performed after sentence segmentation as it eliminates sentence-initial capitalization, which is an important signal for identifying sentence boundaries.

### 3.2 Morphosyntactic analysis

Features from morphological and syntactic analysis are commonly applied in support of entity mention detection. In this work, features identifying the POS and chunk (shallow parsing) tags and word base forms (lemmas) are created using the GENIA tagger (Tsuruoka *et al.*, 2005) with a model trained on the Penn Treebank Wall Street Journal subset (Marcus *et al.*, 1993), the GENIA Treebank (Tateisi and Tsujii, 2006) and the PennBioIE corpus (Kulick *et al.*, 2004). The tool performs lemmatization using a base form dictionary derived from WordNet (Miller, 1995).

### 3.3 Lexical resources

There is a wealth of lexical and ontological resources that could be used to support anatomical entity mention detection, ranging from simple lists of entities to extensive richly structured ontologies such as the FMA. Owing to the large number of potentially applicable resources, we focus here not on the use and evaluation of individual resources, but rather on two major ‘metaresources’: the Unified Medical Language System (UMLS®) Metathesaurus (Bodenreider, 2004) and the Open Biomedical Ontologies (OBO) Foundry (Smith *et al.*, 2007). We additionally consider the effect of applying resources automatically derived from large corpora of unannotated text. For each resource, we develop a simple mapping from text spans to a set of binary features representing e.g. semantic classes, as described in the following.

#### 3.3.1 UMLS Metathesaurus

The UMLS Metathesaurus is an extensive resource integrating lexical and database resources from over 150 sources, including Medical Subject Headings (MeSH), SNOMED Clinical Terms (CT), International Statistical Classification of Diseases and Related Health Problems, 10th Revision (IDC-10) and many other major vocabularies, and contains in total >5 million names for over a million concepts. The UMLS organizes concepts in its Semantic Network, which provides numerous semantic relations as well as 133 semantic types, such as Amino Acid, Disease, Plant and Tissue. These types are applied to categorize all UMLS concepts and provide, in effect, informal shared upper-level ontology. The National Library of Medicine (NLM)® has also developed an associated tool, MetaMap (Aronson, 2001), capable of detecting mentions of UMLS concepts in text. The tool is applied in MEDLINE indexing and various other tasks (Aronson and Lang, 2010). MetaMap performs limited syntactic analysis and disambiguation to improve its concept assignment and can thus provide a better mapping of text to UMLS than naive matching against UMLS vocabularies.

Both standalone use of the UMLS Metathesaurus as well as its application via MetaMap tagging have obvious potential benefits for anatomical entity mention detection. In a previous study of anatomical entity mention tagging, we found a substantial benefit from using MetaMap for feature generation (Ohta *et al.*, 2012). However, there are challenges to the use of the UMLS Metathesaurus and MetaMap in redistributable tools such as AnatomyTagger. First, the full MetaMap distribution requires significant amounts of disk space, memory and processing power to run, and its inclusion would substantially increase the computational requirements of the tagger. Further, both MetaMap and many of the UMLS Metathesaurus sources have licenses that make it impossible to create distributable tools directly incorporating these resources. Thus, instead of incorporating these resources directly, we make use of the MetaMapped Medline® data (http://skr.nlm.nih.gov/resource/MetaMappedBaselineInfo.shtml), a distribution of the output of MetaMap run on the entire PubMed baseline distribution provided by the NLM. We processed these data by first using the mm_print12 tool (http://metamap.nlm.nih.gov/#MetaMapPrint) to convert the MetaMap prolog-like output to extensible mark-up language, which we then parsed to determine semantic class assignments to each string. We then extracted for each string the set of types that were associated with that string in at least half of the cases in which the string appeared in the data to generate a comparatively small number of text string-semantic class pairs as a separate dictionary that can be applied in tagging.

#### 3.3.2 OBO resources

The OBO consortium aims to develop orthogonal, mutually interoperative, openly available biomedical domain ontologies. The OBO Foundry (http://www.obofoundry.org/) collects related resources, and as of this writing lists 115 ontologies, of which 40 involve the domain ‘anatomy’. These range from resources covering the anatomy of individual species, such as FMA (human) to resources such as the Plant Ontology covering an entire kingdom (Cooper *et al.*, 2013), and include two major species-independent resources focusing on specific levels of biological organization: the cellular component subontology of the Gene Ontology (Ashburner *et al.*, 2000) and the Cell Ontology (Meehan *et al.*, 2011).

**Fig. 3.**
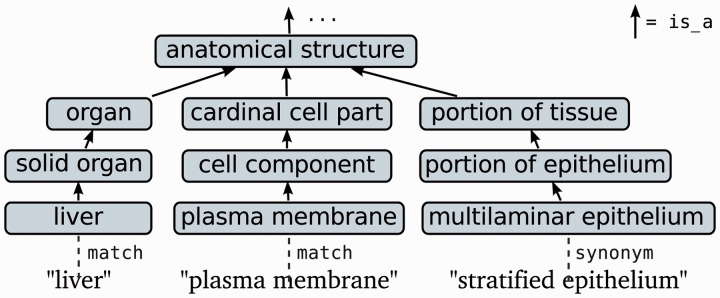
Term matching and classification using OBO resources. Example simplified from FMA; figure modified from Pyysalo *et al.* (2011)

Many of the OBO anatomy-related resources could be used to generate a rich array of features based on information such as the part-of or develops-from relations provided by some ontologies, but as the resources vary greatly in such aspects, we focus on features generated based on basic information present in almost all of the ontologies: entity names and synonyms and their mutual is-a relations. Names and synonyms allow anatomical entity mention candidates to be easily identified using a dictionary lookup approach, and is-a relations allow the likely types of these mentions to be determined (Fig. 3).

We derived a mapping from text to upper-level ontological categories from OBO following an approach we previously introduced for anatomical term classification (Pyysalo *et al.*, 2011). In brief, we first selected from OBO a set of non-redundant anatomy-domain resources with non-trivial is-a structure, giving a set of 26 ontologies (see Supplementary Material). For each term in these ontologies, we follow is-a relations to identify the relevant upper-level ontological categories comparable with the targeted types (Table 1), mapping explicitly to corresponding CARO terms when possible. We then extract term names and synonyms together with these upper-level categories, and further apply the NLM Lexical Variant Generator (McCray *et al.*, 1994) to add e.g. spelling and pluralization variants to the forms found in the OBO resources. Finally, to reduce the dimensionality and sparseness of the feature set, we selected the most frequent 100 upper-level categories in the data and mapped the remaining types to ‘other’.

#### 3.3.3 Word representations

Despite extensive efforts to annotate corpora and create lexicons and ontological resources, the scope of the literature continues to dwarf that of manually curated resources. The availability of a large body of unannotated domain texts offers many opportunities to support also supervised learning. One successful approach is to induce *word representations* that can then be used to introduce additional features for supervised learning. Associating each word with an alternate representation (e.g. a vector) allows non-binary word similarities to be defined, which can in turn alleviate sparseness issues in supervised learning, for example by providing information on the meaning of words not appearing in annotated data. A variety of methods for inducing such representations from unannotated text have been proposed (Brown *et al.*, 1992; Collobert and Weston, 2008; Mnih and Hinton, 2009). These methods draw in one way or another on the observation that words are known by the company they keep (Firth, 1957), i.e. that words appearing in similar contexts tend to have similar meanings.

A number of word representations have been evaluated also in the specific context of supervised sequential labeling tasks such as entity mention detection (Turian *et al.*, 2010). In our recent study of word representations and their domain dependency (Stenetorp *et al.*, 2012b), we found that Brown clusters (Brown *et al.*, 1992) induced on texts from the same domain as annotated resources show clear benefits for various mention recognition tasks. The Brown cluster representation assigns each word a simple bit-string representing a position in a hard hierarchical clustering. In addition to offering competitive performance, the representation is thus sparse, allowing both word-cluster mappings and feature vectors including Brown cluster-derived features to be stored compactly. We thus chose to focus on Brown clusters in the present study. We evaluate performance with two sets of brown clusters: the clusters induced by Turian *et al.* (2010) on newswire texts and newly induced clusters following the approach of Stenetorp *et al.* on a random sample of 500 000 biomedical publication abstracts. Following previous work, we induce and compare clusterings with the cluster number 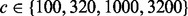 and introduce for each word new features consisting of the 4-, 6- and 10-digit prefixes of its cluster identifier.

#### 3.3.4 Feature generation with lexical resources

The NERsuite toolkit includes an efficient tagger for augmenting feature sets based on mappings from text spans to binary features, operating on either a token or phrase (maximal span) basis. Although this dictionary tagger can be used also for the anatomical entity mention recognition task with the resources described in the preceding sections, preliminary experiments indicated that some aspects of its implementation were not ideal for the task. First, the tagger implemented only leftmost-longest matching: when multiple candidate spans overlap, only the longest of the leftmost spans is tagged. This is potentially problematic when using broad-coverage resources such as UMLS, where longer non-relevant expressions (e.g. ‘growth of cells’) could block the tagging of relevant embedded ones (e.g. ‘cells’). Second, the implementation included a POS-based filter that required each span to include at least one noun (word with a POS tag starting with ‘NN’), which is reasonable for named entity recognition but not optimal for resources involving also other expressions. To address these issues, we modified the NERsuite dictionary tagger to support also a tagging mode where all candidate mentions are marked and to allow flexible configuration of the POS filter. We explore combinations of these options in development test experiments.

### 3.4 Feature representation

The features presented in the previous sections are generated for each token and can be combined in various ways to create a full feature representation for machine learning. As is customary in feature representations for sequential labeling, features are generated for the ‘focus’ token as well as for surrounding tokens in a small window, in our experiments consisting of the two preceding and two following tokens. The original surface form, normalized form (lowercased, etc), lemma, POS tag, chunk tag and lexical resource (dictionary) matching-derived features are generated in standalone and bigram combinations, and token internal structure is captured by character-based *n*-gram and orthography features. The feature representation is presented in full detail in the Supplementary Material.

### 3.5 Non-local features

Sequential tagging setups typically directly provide information from only limited local context to the machine learning method. Although highly effective and sufficient for competitive performance at many tasks, local features cannot capture a number of intuitively informative properties of text, such as that all mentions of a word in a specific discourse tend to carry the same sense (Gale *et al.*, 1992). To allow the tagger to benefit from non-local information, we apply a two-stage tagging strategy inspired by Krishnan and Manning (2006) where a standard model is first applied to create a first-stage tagging that is then analyzed to create additional document-level features for a separately trained second-stage model that produces the final predictions. For the second-stage tagging, we extend the standard feature set used for the first-stage tagging with the following non-local features for each token, based on all identical tokens in the document: (i) most frequent tag, (ii) most frequent non-‘O’tag and (iii) first tag in the document. These features are intended to allow the tagger to e.g. benefit from unambiguous mentions in context to disambiguate ambiguous ones. To train the second-stage model, we create a dataset that incorporates non-local features by training 10 different first-stage models in a cross-validation setup, using each to predict the additional features for 10% of the data.

### 3.6 Parameter settings

In initial experiments, we observed that regardless of the settings of the regularization parameters for CRF training, the resulting models systematically showed high-precision, low-recall results. This reflects a frequent phenomenon where machine learning models tend to favor the majority class—here ‘O’, for outside—that is reasonable when optimizing for accuracy, but may not correspond to user preference and can lead to suboptimal performance in terms of other metrics, such as entity mention detection F-score. To address this issue, a new feature allowing *label bias* weights to be set in decoding was implemented in CRFsuite and NERsuite, following the approach of Minkov *et al.* (2006). By assigning lower weights to ‘O’ labels or higher weights to ‘B’ or ‘I’ labels, decoding can be fine-tuned toward producing more entity mentions, without requiring retraining of the model.

### 3.7 Corpus

For the training and evaluation of our methods, we created the extended Anatomical Entity Mention (AnatEM) corpus. This corpus builds in part on two previously introduced resources, the AnEM and Multi-Level Event Extraction (MLEE) corpora. The Anatomical Entity Mention (AnEM) corpus was introduced by Ohta *et al.* (2012) and consists of 500 randomly selected PubMed abstracts and full-text extracts (sections and captions) annotated for anatomical entity mentions using the same scope definitions and 11 of the 12 types applied in this work, with the exception that AnEM lacks the Pathological formation/Cancer distinction (Table 1). The MLEE corpus, introduced by Pyysalo *et al.* (2012a), consists of 262 PubMed abstracts on the molecular mechanisms of cancer, specifically relating to angiogenesis. Among its annotations, MLEE is annotated for anatomical entities following the AnEM task setting.

To create the AnatEM corpus, we initially combined the anatomical entity annotations of the AnEM and MLEE corpora, and then proceeded to perform manual annotation using the brat tool (Stenetorp *et al.*, 2012a) for an additional set of 100 documents following the selection criteria of the former and 350 documents following those of the latter, for a selection of topics related to cancer. The resulting corpus thus consists of 1212 documents, 600 of which are drawn randomly from abstracts and full texts as in AnEM, and the remaining 612 are a targeted selection of PubMed abstracts relating to the molecular mechanisms of cancer. We also evaluated all Pathological formation annotations in the previously introduced corpora to introduce the Cancer type, and performed several rounds of consistency checking, supported by automatic tools developed for the task. Table 2 presents overall statistics of the AnatEM corpus, contrasting it with a number of previously released corpora containing annotations for at least some anatomical entity types: the Colorado Richly Annotated Full Text (CRAFT) corpus (Bada *et al.*, 2012), the CellFinder corpus (Neves *et al.*, 2012) and the Joint Workshop on Natural Language Processing in Biomedicine and its Applications (JNLPBA) corpus (Kim *et al.*, 2004).

**Table 2.** Corpus statistics

Name	Entities	Tokens	Documents	Sources	Annotated anatomical entity types
AnatEM	13 701	245 448	1212	Abstracts, full text extracts	12 anatomical entity types (Table 1)
AnEM	3135	91 420	500	Abstracts, full text extracts	Same as AnatEM, but no pathological formation/cancer distinction
MLEE	3599	56 588	262	Abstracts	Same as AnatEM, but no pathological formation/cancer distinction
CellFinder	3667	55 362	10	Full texts	Cell component, cell, generic anatomy type
CRAFT*^a^*	14 248	587 299	67	Full texts	Cell component, cell
JNLPBA	12 969	522 869	2404	Abstracts	Cell

*Note*: Annotated types and entity mention counts shown for anatomical entities as defined in Section 2.1. *^a^*Statistics for publicly available part of corpus.

### 3.8 Experimental setup

The AnatEM corpus was split into separate training, development and test sets, following the existing divisions of the AnEM and MLEE corpora for documents drawn from these resources and dividing the remaining documents randomly to create a 50%/17%/33% train/devel/test split.

The selection of the regularization and label bias parameters (Section 3.6), fine-tuning of the feature representation and other comparable detailed settings were performed by evaluation on the development set only. The L2 regularization coefficient was selected from 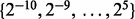 and the label bias parameter for begin (‘B’) labels from 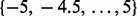. The optimal feature representation and parameter settings were then applied in a single set of final experiments where the method was trained on the combination of the training and development sets and tested on the test set documents. Correspondingly, except for the final evaluation results, all results are for optimal parameter values on the development set, and a degree of overfitting is thus expected.

Results are reported using the precision and recall metrics and their harmonic mean, the *F*_1_-score. These metrics are calculated on the entity mention level. We apply right boundary match as the primary evaluation criterion throughout, following Ohta *et al.* (2012). Types are required to match exactly also in the multiclass setting.

## 4 RESULTS AND DISCUSSION

### 4.1 Evaluation on development data

The results of the evaluation of the various enhancements to the baseline tagger are summarized in Table 3.

**Table 3.** Development test results (F-scores)

Method	Single-class	Multiclass
Baseline	86.93	81.23
Truecasing	87.12	81.45
Non-local features	87.81	81.82
UMLS, tokens	89.07	82.79
UMLS, longest phrases	88.57	82.64
UMLS, all phrases	89.65	83.50
OBO, tokens	87.58	81.76
OBO, longest phrases	88.81	82.56
OBO, all phrases	88.40	82.44
Brown, news, c = 100	87.20	80.92
Brown, news, c = 320	87.71	81.23
Brown, news, c = 1000	86.58	80.68
Brown, news, c = 3200	87.11	80.80
Brown, bio, c = 100	87.44	81.67
Brown, bio, c = 320	89.56	82.03
Brown, bio, c = 1000	88.94	81.78
Brown, bio, c = 3200	88.55	81.33

We find that truecasing gives only a modest positive effect, possibly related to the inclusion of lowercased variants of tokens in the base feature set. However, as this limited benefit was found also in combination with other approaches (data not shown), we opted to include truecasing in the final tagger. By contrast, we find a substantial benefit from the incorporation of non-local features through two-stage processing, giving a 7% reduction in F-score error in the single-class setting and 3% for the multiclass setting.

The evaluation of the effect of incorporating features based on tagging against UMLS- and OBO-derived resources shows that both provide notable advantages for anatomical entity mention detection. The highest performance is found for UMLS when tagging all phrases, which gives an impressive 21% relative reduction in error in the single-class setting and a 12% reduction in the multiclass setting. Although phrase-based tagging is found to be more effective than token-based tagging for both resources, the two resources show mixed effects regarding the benefits of tagging longest phrases only versus all phrases, with OBO favoring the former and UMLS the latter. This effect may be explained in part by the presence of terms not related to anatomy in the UMLS-derived resource, whereas the OBO-derived resource only contains anatomy-relevant terms.

The results of evaluation using Brown clusters with various numbers of clusters (*c*) indicate that the use of clusters induced using out-of-domain texts (news) does not benefit tagging performance, and may even affect it negatively. For Brown clusters induced on in-domain data (bio), we find that all of the sets provide some benefit, with a notably more positive effect on single-class than multiclass tagging. Contrary to expectation, there is no clear indication that larger numbers of clusters would be more beneficial. The domain dependence of Brown cluster features observed here agrees with our previous findings using a different machine learning method (Stenetorp *et al.*, 2012b). The current evaluation confirms that the benefit of using Brown cluster-derived features is strongly domain-dependent also for CRF-based entity mention tagging.

### 4.2 Comparative evaluation on test data

For the final evaluation on the held-out test data, we trained the full AnatomyTagger system using the best settings identified in the development experiments presented in the preceding sections. As points of comparison, we consider the dictionary-based tagger introduced in the BioContext project (Gerner *et al.*, 2012) and MetaMap, restricted to tagging types relevant to anatomy (following Ohta *et al.*, 2012). We additionally train and apply three state-of-the-art machine learning-based taggers on these data: the original NERsuite system that AnatomyTagger is based on, the most recent version of the Illinois NER system (Ratinov and Roth, 2009) and the recently introduced Gimli system, which was reported to outperform all other available biomedical domain taggers on two established reference tasks (Campos *et al.*, 2013). All machine learning-based taggers were trained on the combination of the AnatEM corpus training and development sets.

Table 4 summarizes the results of the comparative evaluation. Please note that Gimli does not support the entity types applied here and was only evaluated by replacing types with a single type recognized by the system. Detailed results and error analysis are presented in supplementary Tables 6–9. Interestingly, we find that for these data, the unmodified NERsuite already outperforms both the general-domain Illinois tagger as well as the biomedical domain tagger Gimli. This may reflect in part the specific focus of Gimli on biomolecular entity recognition. The full AnatomyTagger further outperforms the unmodified NERsuite, showing an additional 22% reduction in error for the single-class and 10% reduction for the multiclass case. We find the performance of the developed system on this corpus highly promising, in particular in light of the earlier evaluation results of Ohta *et al.* (2012), who reported a best set of results of 78.34% F-score for single-class and 68.37% for multiclass in a comparable task. Our results indicate that anatomical entity mention recognition can be performed at levels of reliability comparable with those of well-established domain tasks such as gene mention recognition (Smith *et al.*, 2008).

**Table 4.** Comparative evaluation on test data (F-scores)

Method	Single-class	Multiclass
BioContext	38.97	—
MetaMap	67.34	—
Illinois	81.01	75.22
Gimli	86.75	—
NERsuite	89.20	83.50
AnatomyTagger	**91.61**	**85.11**

Highest results highlighted in bold.

### 4.3 Literature-scale application

To apply the tagger at large scale, we created an implementation of the tagger in UIMA framework (Ferrucci and Lally, 2004), integrated in the U-Compare/Argo system (Kano *et al.*, 2009; Rak *et al.*, 2012). The system was applied in the University of Manchester HTCondor high-throughput computing system (3000 cores) to tag all 606 389 PubMed Central® Open Access articles. The processing averaged usage of ∼100 cores and required a week to complete. The application resulted in the recognition of 48 470 652 anatomical entity mentions in total. The statistics of tagged types are shown in Table 5 and further details on the tagged entity mentions are provided in Supplementary Material. Based on these results, we estimate that there are on average 80 anatomical entity mentions in a full-text document, and 26 mentions per 1000 words. The full automatically annotated dataset is provided in the simple tab-separated values-style standoff format first introduced in the BioNLP Shared Task (Kim *et al.*, 2011) and used in many domain tools and resources.

**Table 5.** Tagged entity counts in PMC OA documents

Type	Count
Organism subdivision	2 429 093
Anatomical system	511 191
Organ	5 101 355
Multi-tissue structure	6 855 622
Tissue	2 541 481
Cell	16 062 208
Developing anatomic structure	478 429
Cellular component	4 824 697
Organism substance	3 717 117
Immaterial anatomic entity	699 962
Pathological formation	702 526
Cancer	4 546 971
Total	48 470 652

### 4.4 Related work

Although a wealth of general machine learning methods could be applied to train anatomical entity mention recognition systems, we are not aware of previous efforts to develop such a system dedicated to anatomical entities, a point noted also by Campos *et al.* (2012) in their recent survey of domain tools. Anatomical entities fall broadly within the scope of various general tagging methods based on matching against lexical resources, such as MetaMap (Aronson, 2001) and the NCBO annotator (Jonquet *et al.*, 2009). A related approach drawing on matching against dictionaries derived from OBO was also applied by Gerner *et al.* (2012), the only previous effort involving large-scale anatomical entity mention detection that we are aware of. However, as shown by our experiments, generic dictionary matching-based methods tend to perform weakly compared with dedicated machine learning methods trained on sufficient resources, motivating the development of a dedicated tagger.

## 5 CONCLUSION

We have presented AnatomyTagger, a system for the recognition of anatomical entity mentions in free text. The machine learning-based tagger integrates a variety of techniques shown to benefit tagging performance, including manually curated lexical resources, word representations induced from unannotated text, statistical truecasing and non-local features. Evaluation demonstrated that all techniques incorporated into AnatomyTagger were beneficial compared with a strong baseline tagger and that the final integrated system outperforms previously introduced methods for comparable tasks.

We then applied a UIMA implementation of the tagging pipeline to the entire open access biomedical literature—over 600 000 full-text documents—to create an automatically tagged resource of >48 million entity mentions. We are currently applying the method to the entire Europe PubMed Central literature and integrating the results into the Europe PubMed Central search and analytics services.

All tools and resources introduced in this work are available under open licenses from http://nactem.ac.uk/anatomytagger.

## Supplementary Material

Supplementary Data
