# Supplementary material for: Anatomical entity mention recognition at literature scale
Source: Bioinformatics. 2013 Oct 25;30(6):868–75. doi: 10.1093/bioinformatics/btt580 (PMC3957068; doi:10.1093/bioinformatics/btt580)
Supplement: Supplementary Data [file supp_30_6_868__index.html]

Anatomical Entity Mention Recognition at Literature Scale — Anatomical entity mention recognition at literature scale — Anatomical entity mention recognition at literature scale — Supplementary Data 

# Anatomical entity mention recognition at literature scale

## Supplementary Data

files

**Files in this Data Supplement:**

- Supplementary Data - pdf file
