# Supplementary material for: Anatomical entity mention recognition at literature scale
Source: Bioinformatics. 2013 Oct 25;30(6):868–75. doi: 10.1093/bioinformatics/btt580 (PMC3957068; doi:10.1093/bioinformatics/btt580)
Supplement: Supplementary Data [file supp_btt580_anemtag-supp.pdf]

Supplementary Information

Anatomical Entity Mention Recognition at Literature Scale

Sampo Pyysalo and Sophia Ananiadou

This document provides supplementary information for the manuscript *Anatomical Entity Mention Recognition at Literature Scale*.

1 ONTOLOGICAL BASIS

The following Common Anatomy Reference Ontology (CARO) and Foundational Model of Anatomy (FMA) definitions delimit the primary scope of the annotation. The annotation targets are mentions of anatomical entities.

`anatomical entity`$_{\text{CARO}}$
Biological entity that is either an individual member of a biological species or constitutes the structural organization of an individual member of a biological species.

The primary subcategory of anatomical entity mentions are anatomical structures.

`anatomical structure`$_{\text{CARO}}$
Material anatomical entity that has inherent 3D shape and is generated by coordinated expression of the organism's own genome.

Anatomical structures are subdivided into comprehensive, nonoverlapping categories by granularity. We exclude from the scope of the annotation mentions of biological macromolecules and whole organisms.

`Biological macromolecule`$_{\text{FMA}}$
Anatomical structure which has as its parts one or more ordered aggregates of nucleotide, amino acid fatty acid or sugar molecules bonded to one another.

`multi-cellular organism`$_{\text{CARO}}$
Anatomical structure that is an individual member of a species and consists of more than one cell.

To avoid overlap with organism name recognition tasks, we also exclude from annotation mentions of single cell organism names. We refer to Section 2.1 of the primary manuscript and the CARO and FMA ontologies for further informaion.

2 NERSUITE FEATURES

Table 1 details the features applied by NERsuite. Please refer to the paper for details on the feature category definitions.

Table 1. Features for entity detection

Class	Definition
Token	$\{w_{t-2}, .., w_{t+2}\}$, $\{w_{t-2,t-1}, .., w_{t+1,t+2}\}$, $\{\bar{w}_{t-2}, .., \bar{w}_{t+2}\}$, $\{\bar{w}_{t-2,t-1}, .., \bar{w}_{t+1,t+2}\}$
Lemma	$\{l_{t-2}, .., l_{t+2}\}$, $\{l_{t-2,t-1}, .., l_{t+1,t+2}\}$, $\{\bar{l}_{t-2}, .., \bar{l}_{t+2}\}$, $\{\bar{l}_{t-2,t-1}, .., \bar{l}_{t+1,t+2}\}$
POS	$\{p_{t-2}, .., p_{t+2}\}$, $\{p_{t-2,t-1}, .., p_{t+1,t+2}\}$
Lemma & POS	$\{l_{t-2}p_{t-2}, .., l_{t+2}p_{t+2}\}$, $\{l_{t-2,t-1}p_{t-2,t-1}, .., l_{t+1,t+2}p_{t+1,t+2}\}$
Chunk	$\{c_t, w_{t\_last}, \bar{w}_{t\_last}, the_{lhs}\}$
Character	Character 2,3,4-grams of w_t
Orthography	All capitalized, all numbers, contain Greek letters, etc., following Lee *et al.* (2004)
Dictionary	$\{d_{t-2}, .., d_{t+2}\}$, $\{d_{t-2,t-1}, .., d_{t+1,t+2}\}$ $\{d_{t-2}w_{t-2}, .., d_{t+2}w_{t-2}\}$,<br>$\{d_{t-2,t-1}w_{t-2,t-1}, .., d_{t+1,t+2}w_{t+1,t+2}\}$

Symbols used: w_t: token text; l_t: lemma; p_t: POS tag; c_t: chunk tag; w_{t_last}: last word of current chunk; the_{lhs}: token "the" present in current chunk; d_t: dictionary matching result; $\bar{x}$: normalized form of x.

Dictionary features are only generated if matching against dictionaries has been performed for input data. NERsuite is not distributed with any dictionaries and does not perform matching against dictionaries automatically. Dictionaries need to be provided by the user and dictionary matching performed separately, e.g. using the `nersuite_dictionary_tagger` tool distributed with NERsuite.

Note that while extensions such as truecasing and non-local features (see Section 3 of the manuscript) are incorporated into the NERsuite feature representation, they are not part of the standard NERsuite implementation.

3 APPLICATION OF METAMAP AND UMLS® RESOURCES

The MetaMapped Medline® data[1] applied to create the UMLS-based dictionary was created by NLM® using MetaMap with the command

```
metamap11v2 -Z 1112 -qE -Q 4
```

(see `http://metamap.nlm.nih.gov/MM11_Usage.shtml` for information on MetaMap parameters.)

For MetaMap-based anatomical entity mention tagging, we applied MetaMap with the command

```
metamap12 -J acab,anab,anst,bdsu,bdsy,blor,bpoc,bsoj,celc,cell,emst,ffas,neop,tisu
```

Here, the `-J` argument constrains tagging to the following subset of UMLS classes

Table 2. Tagged UMLS semantic types

`acab`	Acquired Abnormality
`anab`	Anatomical Abnormality
`anst`	Anatomical Structure
`bdsu`	Body Substance
`bdsy`	Body System
`blor`	Body Location or Region
`bpoc`	Body Part, Organ, or Organ Component
`bsoj`	Body Space or Junction
`celc`	Cell Component
`cell`	Cell
`emst`	Embryonic Structure
`ffas`	Fully Formed Anatomical Structure
`neop`	Neoplastic Process
`tisu`	Tissue

(see `http://mmtx.nlm.nih.gov/MMTx/semanticTypes.shtml` for the definitions of the UMLS semantic types)

[1] `http://skr.nlm.nih.gov/resource/MetaMappedBaselineInfo.shtml`

4 APPLICATION OF OBO FOUNDRY RESOURCES

Table 3 lists the selected OBO Foundry "anatomy" domain resources from which the OBO dictionary was extracted.

Table 3. Applied OBO anatomy resources

Resource name (prefix)	Size
Foundational Model of Anatomy (**FMA**)	78977
Drosophila gross anatomy (**FBbt**)	7338
C. elegans gross anatomy (**WBbt**)	7132
Uber anatomy ontology (**UBERON**)	6339
BRENDA tissue / enzyme source (**BTO**)	5139
Teleost Anatomy Ontology (**TAO**)	3038
Gene Ontology* Cellular component subontology (**GO-CC**)	2982
Mouse adult gross anatomy (**MA**)	2982
Zebrafish anatomy and development (**ZFA**)	2708
Human developmental anatomy, abstract version, v2 (**EHDAA2**)	2464
Hymenoptera Anatomy Ontology (**HAO**)	1903
Cell type (**CL**)	1882
Mosquito gross anatomy (**TGMA**)	1861
Amphibian gross anatomy (**AAO**)	1603
Plant Ontology (**PO**)	1270
Subcellular anatomy ontology (**SAO**)	826
Xenopus anatomy and development (**XAO**)	817
Tick gross anatomy (**TADS**)	628
Spider Ontology (**SPD**)	577
Vertebrate Anatomy Ontology (**VAO**)	139
Dictyostelium discoideum anatomy (**DDANAT**)	138
Anatomical Entity Ontology (**AEO**)	137
Dendritic cell (**DC_CL**)	113
Bilateria anatomy (**BILA**)	105
Fungal gross anatomy (**FAO**)	81
Common Anatomy Reference Ontology (**CARO**)	48

5 CORPUS ANNOTATION STATISTICS

Table 4 presents the statistics of the AnatEM corpus by annotated entity type.

Table 4. Corpus annotation statistics

Type	Count
Organism subdivision	336
Anatomical system	112
Organ	863
Multi-tissue structure	1695
Tissue	843
Cell	4521
Developing anatomical structure	100
Cellular component	829
Organism substance	685
Immaterial anatomical entity	261
Pathological formation	391
Cancer	3065

6 EVALUATION WITH DIFFERENT MATCHING CRITERIA

Tables 5–8 present detailed results for the comparative evaluation on test data for various matching criteria.

Table 5. Evaluation on test data, exact matching criterion (precision / recall / F-score)

Method	Single-class			Multi-class		
BioContext	56.2	22.4	32.1		-	
MetaMap	51.5	58.1	54.6		-	
Illinois	83.1	65.2	73.1	77.5	60.8	68.1
Gimli	87.3	75.1	80.8		-	
NERsuite	87.1	77.9	82.2	84.1	72.1	77.7
AnatomyTagger	88.5	82.6	**85.5**	84.1	75.4	**79.5**

Table 6. Evaluation on test data, left boundary matching criterion (precision / recall / F-score)

Method	Single-class			Multi-class		
BioContext	68.3	27.2	38.9		-	
MetaMap	60.3	67.6	63.8		-	
Illinois	88.5	69.4	77.8	79.6	62.4	69.9
Gimli	90.5	77.8	83.7		-	
NERsuite	89.8	80.3	84.8	85.7	73.4	79.1
AnatomyTagger	90.7	84.8	**87.6**	85.4	76.5	**80.7**

Table 7. Evaluation on test data, right boundary matching criterion (precision / recall / F-score)

Method	Single-class			Multi-class		
BioContext	68.3	27.3	39.0		-	
MetaMap	63.8	71.1	67.3		-	
Illinois	92.2	72.2	81.0	85.6	67.1	75.2
Gimli	93.8	80.6	86.7		-	
NERsuite	94.4	84.5	89.2	90.4	77.5	83.5
AnatomyTagger	94.8	88.6	**91.6**	90.0	80.7	**85.1**

All methods other than MetaMap show higher precision than recall for all criteria, with BioContext performance in particular being limited by low recall. F-scores increase in cases by over 10% points when moving from exact matching to overlap matching, indicating that differences in tagged and annotated entity boundaries are a frequent source of error when evaluating with strict matching. Regardless of the matching criteria applied, the ranking of the methods by F-score remains unchanged.

Table 8. Evaluation on test data, overlap matching criterion (precision / recall / F-score)

Method	Single-class			Multi-class		
BioContext	84.6	32.5	46.9		-	
MetaMap	73.7	76.9	75.3		-	
Illinois	98.0	75.7	85.4	87.6	68.5	76.9
Gimli	96.9	83.4	89.7		-	
NERsuite	96.9	86.8	91.5	92.0	78.8	84.9
AnatomyTagger	96.8	90.6	**93.6**	91.4	81.8	**86.3**

7 EVALUATION RESULTS BY DOMAIN

Table 9 shows evaluation results separately the two subdomains of the literature from which the AnatEM corpus documents have been drawn: random biomedical publications, and abstracts of publications regarding cancer. Please refer to Section 3.7 in the main manuscript for further information on the corpus construction.

Table 9. Evaluation on test data for randomly drawn and cancer domain documents, right boundary matching criterion (F-scores). Overall results repeated for reference.

	Random		Cancer		Overall	
Method	Single-class	Multi-class	Single-class	Multi-class	Single-class	Multi-class
BioContext	49.5	-	33.6	-	39.0	-
MetaMap	62.5	-	69.5	-	67.3	-
Illinois	69.6	62.6	85.4	80.1	81.0	75.2
Gimli	75.3	-	91.3	-	86.7	-
NERsuite	80.7	72.3	92.7	87.9	89.2	83.5
AnatomyTagger	**85.1**	**76.6**	**94.4**	**88.6**	**91.6**	**85.1**

The two methods based on dictionary matching perform better on random documents, perhaps reflecting particular challenges on cancer domain documents. As expected, the machine learning-based methods show better performance on restricted domain (cancer) documents than on general-domain (random) documents, reflecting the sparsity and variety of examples in the latter. Despite the different strengths, the ranking of the methods remains the same as in the overall evaluation for both subsets of the data.

8 EVALUATION RESULTS BY ENTITY TYPE

Tables 10–12 show test set evaluation results by entity type for the methods that could be trained to perform multi-class entity mention detection. Overlap matching criteria are applied to reduce the effects of boundary errors on evaluated performance.

Table 10. Illinois tagger evaluation on test data, overlap matching criterion (precision / recall / F-score)

Type	Prec.	Recall	F-score
ANATOMICAL SYSTEM	3.9	20.5	6.6
CANCER	84.1	76.2	80.0
CELL	88.6	75.9	81.8
CELLULAR COMPONENT	41.2	27.4	32.9
DEVELOPING ANATOMICAL STRUCTURE	17.9	28.3	21.9
IMMATERIAL ANATOMICAL ENTITY	14.2	23.4	17.6
MULTI-TISSUE STRUCTURE	42.4	41.1	41.7
ORGAN	45.1	39.4	42.0
ORGANISM SUBDIVISION	10.9	13.0	11.8
ORGANISM SUBSTANCE	61.7	42.8	50.5
PATHOLOGICAL FORMATION	16.7	20.8	18.6
TISSUE	25.2	36.9	29.9

Table 11. NERsuite evaluation on test data, overlap matching criterion (precision / recall / F-score)

Type	Prec.	Recall	F-score
ANATOMICAL SYSTEM	7.5	19.2	10.8
CANCER	92.5	80.3	86.0
CELL	94.6	81.7	87.6
CELLULAR COMPONENT	65.6	45.2	53.5
DEVELOPING ANATOMICAL STRUCTURE	27.4	26.7	27.0
IMMATERIAL ANATOMICAL ENTITY	16.5	25.4	20.0
MULTI-TISSUE STRUCTURE	59.1	45.9	51.7
ORGAN	62.8	63.8	63.3
ORGANISM SUBDIVISION	13.2	19.3	15.6
ORGANISM SUBSTANCE	80.1	64.4	71.4
PATHOLOGICAL FORMATION	24.1	38.9	29.8
TISSUE	41.0	44.6	42.8

As expected, the performance of the machine learning correlates strongly with the number of examples (Table 4) ranging from very low (6-11% F-score) for rare types such as ANATOMICAL SYSTEM to high (81-91% F-score) for the most common types CELL and CANCER.

Table 12. AnatomyTagger evaluation on test data, overlap matching criterion (precision / recall / F-score)

Type	Prec.	Recall	F-score
Anatomical system	9.1	14.9	11.3
Cancer	94.5	87.9	91.1
Cell	96.5	84.5	90.1
Cellular component	65.2	46.5	54.3
Developing anatomical structure	17.0	30.2	21.8
Immaterial anatomical entity	13.0	34.4	18.8
Multi-tissue structure	58.6	47.9	52.7
Organ	63.4	57.3	60.2
Organism subdivision	19.5	22.4	20.8
Organism substance	81.6	55.3	65.9
Pathological formation	24.7	47.7	32.5
Tissue	38.5	45.2	41.6

9 ANALYSIS OF TAGGING ERRORS

Tables 13–16 show the strings that were most frequently tagged by each method but not annotated as anatomical entity mentions in the corpus (false positives) and the annotated strings that were most frequently not tagged by each system. Overlap matching criteria and single-class evaluation are applied to reduce the effects of boundary and entity typing errors on the analysis.

Table 13. Most frequent false positives and negatives on test data for BioContext

False positive		False negative	
String	Count	String	Count
ST	12	tumor	174
fibroblast	12	cells	134
PS	10	cell	125
PSP	9	tumors	50
HGF	8	cancer	49
band	8	vascular	47
TLX	7	tissue	46
KB	7	serum	44
platelet	6	cellular	42
MR	6	tumour	32

Short, ambiguous abbreviations are a problem for the precision of BioContext, and that the low recall of is primarily caused by not tagging common non-specific mentions of anatomical entities such as *tumor* and *cells*.

Table 14. Most frequent false positives and negatives on test data for MetaMap

False positive		False negative	
String	Count	String	Count
time	62	tumor	25
genetic	56	cells	22
metastasis	53	wound	18
lower	34	SCC	16
medium	26	samples	15
sites	25	Mo	12
process	25	cell	12
tumorigenesis	24	surface	11
vascular endothelial	22	cultures	10
origin	22	cellular	10

MetaMap false positives indicate that a number of unexpected strings match in UMLS with one or more of the semantic classes shown in Table 2 (e.g. *time* as Body Location or Region). Potential issues with semantic class boundaries or class selection is indicated by e.g. the appearance of *metastasis* as a false positive and *tumor* as false negative. As expected, ambiguous words such as *surface* requiring disambiguation based on context represent a challenge for the tagger.

Table 15. Most frequent false positives and negatives on test data for Illinois tagger

False positive		False negative	
String	Count	String	Count
surface	8	growth cone	21
cystic	3	beta-cell	21
anticancer	3	tumor	19
tumour	2	cell	16
thyroid	2	samples	14
platelet	2	Mo	12
nuclear	2	hip	11
neural	2	fetal	10
muscle	2	LE	9
membranes	2	CC-RCC	9

The machine learning-based taggers show fewer clear patterns in their false positives, but share much of the list of most frequent false negatives. The most frequent false negative for all machine learning based systems except AnatomyTagger is one that never appears tagged in the training data. That AnatomyTagger succeeds to tag this string may reflect the use of external resources (dictionaries) in the system, providing additional background knowledge on anatomical entities that is lacking from the other systems. Short, ambiguous abbreviations remain challenging also for the machine learning-based systems.

Table 16. Most frequent false positives and negatives on test data for Gimli

False positive		False negative	
String	Count	String	Count
corticosteroids	8	growth cone	21
surface	5	Mo	12
heminasal aplasia	4	hip	10
food samples	4	CC-RCC	9
cystic	3	LE	8
CCC	3	Ve	7
capsular	3	PF suture	7
anticancer	3	GB	7
stromal cell	2	strain	6
samples	2	samples	6

Table 17. Most frequent false positives and negatives on test data for NERsuite

False positive		False negative	
String	Count	String	Count
surface	5	growth cone	21
heminasal aplasia	4	Mo	12
food samples	4	hip	11
calves	4	LE	9
humoral	3	Ve	7
cortisol	3	strain	7
ceftriaxone	3	PF suture	7
CCC	3	GB	7
anticancer	3	sample	6
venomous	2	PRP	6

Table 18. Most frequent false positives and negatives on test data for AnatomyTagger

False positive		False negative	
String	Count	String	Count
SLAP-2	9	Mo	12
surface	7	LE	9
calves	7	Ve	7
food samples	4	strain	7
CCC	4	PF suture	7
neural network	3	GB	7
junctional particles	3	sample	6
capsular	3	PRP	6
anticancer	3	LCs	6
stromal cell	2	HGF	6

10 ANATOMICAL ENTITY TAGGING STATISTICS

Table 19 provides statistics on the most frequently tagged entity mention strings by entity category.

String	Count
cells	2419631
cell	1714007
cellular	402136
neurons	254017
strains	228474
Cells	214817
macrophages	157422
neuronal	143993
T cells	136330
cell lines	115806

(a) CELL

String	Count
sections	137831
vascular	133246
nodes	99762
node	89721
site	89275
neural	88601
myocardial	84177
cortical	68029
coronary	67895
bone marrow	67678

(b) MULTI-TISSUE STRUCTURE

String	Count
brain	488847
liver	347229
heart	272784
skin	229536
lung	210204
muscle	205761
cardiac	194101
renal	144015
eye	130512
kidney	123728

(c) ORGAN

String	Count
membrane	360308
nuclear	255099
surface	252362
plasmid	204185
mitochondrial	193536
chromosome	183610
chromatin	127510
nucleus	123906
nuclei	113057
mitochondria	108343

(d) CELLULAR COMPONENT

String	Count
tumor	478772
cancer	397611
tumors	178195
breast cancer	178053
tumour	128825
samples	105660
cancers	78289
tumours	66634
HCC	53729
prostate cancer	53366

(e) CANCER

String	Count
blood	630776
serum	512719
samples	348687
plasma	281806
cytoplasmic	109427
extracts	101130
cytoplasm	92070
supernatant	85236
urine	78755
milk	73513

(f) ORGANISM SUBSTANCE

String	Count
tissue	417212
tissues	246654
bone	188998
cartilage	43802
adipose tissue	33811
capillary	31422
epithelial	26652
specimens	26388
endothelium	25346
epithelium	24349

(g) TISSUE

String	Count
body	433504
oral	187260
head	155595
arm	98067
abdominal	72059
neck	62324
knee	60921
hip	59390
breast	56212
hand	53044

(h) ORGANISM SUBDIVISION

String	Count
lesions	115555
lesion	105471
wound	84769
glaucoma	24354
wounds	21702
edema	16558
thrombus	10975
cystic	9778
ulcer	8041
ulcerative	6905

(i) PATHOLOGICAL FORMATION

String	Count
intracellular	229660
extracellular	117927
intraperitoneal	25068
subcutaneous	23864
intracranial	19912
percutaneous	19401
lumen	18902
subcutaneously	18036
intravenously	17818
intraperitoneally	14507

(j) IMMATERIAL ANATOMICAL ENTITY

String	Count
cardiovascular	164327
respiratory	87350
immune system	58628
CNS	36507
central nervous system	35195
nervous system	22003
musculoskeletal	13470
endocrine	8509
neurologic	7272
respiratory tract	7022

(k) ANATOMICAL SYSTEM

String	Count
embryos	180799
embryo	70695
embryonic	59739
eggs	49952
egg	29725
fetus	18063
fetal	17957
fetuses	11759
Embryos	9210
notochord	4044

(l) DEVELOPING ANATOMICAL STRUCTURE

Table 19. Strings most frequently tagged as anatomical entity mentions by type.

REFERENCES

Lee, K.-J. *et al.* (2004). Biomedical named entity recognition using two-phase model based on svms. *J. of Biomedical Informatics*, **37**(6), 436–447.
